# Evaluation of the Georeferencing Accuracy of a Photogrammetric Model Using a Quadrocopter with Onboard GNSS RTK

**DOI:** 10.3390/s20082318

**Published:** 2020-04-18

**Authors:** Martin Štroner, Rudolf Urban, Tomáš Reindl, Jan Seidl, Josef Brouček

**Affiliations:** Department of Special Geodesy, Faculty of Civil Engineering, Czech Technical University in Prague, Thákurova 7, 166 29 Prague, Czech Republic; rudolf.urban@fsv.cvut.cz (R.U.); tomas.reindl@fsv.cvut.cz (T.R.); jan.seidl@fsv.cvut.cz (J.S.); josef.broucek@fsv.cvut.cz (J.B.)

**Keywords:** accuracy, onboard GNSS RTK, UAV

## Abstract

Using a GNSS RTK (Global Navigation Satellite System Real Time Kinematic) -equipped unmanned aerial vehicle (UAV) could greatly simplify the construction of highly accurate digital models through SfM (Structure from Motion) photogrammetry, possibly even avoiding the need for ground control points (GCPs). As previous studies on this topic were mostly performed using fixed-wing UAVs, this study aimed to investigate the results achievable by a quadrocopter (DJI Phantom 4 RTK). Three image acquisition flights were performed for two sites of a different character (urban and rural) along with three calculation variants for each flight: georeferencing using ground-surveyed GCPs only, onboard GNSS RTK only, and a combination thereof. The combined and GNSS RTK methods provided the best results (at the expected level of accuracy of 1–2 GSD (Ground Sample Distance)) for both the vertical and horizontal components. The horizontal positioning was also accurate when georeferencing directly based on the onboard GNSS RTK; the vertical component, however, can be (especially where the terrain is difficult for SfM evaluation) burdened with relatively high systematic errors. This problem was caused by the incorrect identification of the interior orientation parameters calculated, as is customary for non-metric cameras, together with bundle adjustment. This problem could be resolved by using a small number of GCPs (at least one) or quality camera pre-calibration.

## 1. Introduction

Photogrammetry is presently a widely used method, particularly in combination with the Structure from Motion (SfM) technique. This combination allows the capture of surfaces in the form of point clouds (and derived products such as meshes, orthophotos, etc.), facilitating a relatively high degree of automation. The accuracy of multi-rotor UAV (Unmanned Aerial Vehicle) products is very important and is addressed in several studies [1,2,3,4,5,6].

Fixed-wing unmanned aerial vehicles (UAVs), such as eBee, mounted with a GNSS RTK (Global Navigation Satellite System, Real-Time Kinematic) module capable of determining the position of the drone with an accuracy of a few centimeters, have been available for some time now. Since last year, however, moderately priced UAV quadrocopters with GNSS RTK technology have come to the market. The pioneer in this area is DJI Phantom 4 RTK (approximately 7500 Eur).

From the perspective of workflow effectiveness, the idea of relying purely on GNSS RTK technology without the need for geodetic surveys (i.e., calculation of the photogrammetric model using only the imagery coordinates for georeferencing) is very attractive. Given the principle of the calculation where many variables (some of them mutually dependent) are used, it is, however, necessary to verify if georeferencing using only internal data can yield results of sufficient accuracy or whether the use of ground control points (GCPs) is necessary. The declared accuracy of the GNSS RTK receiver in the Czech Republic is approximately 0.025 m in the horizontal plane, while the vertical coordination is approximately 50% higher (0.04 m). 

In geodesy, the use of GCPs is required to verify the accuracy of measurements. However, alternative appropriate solutions are preferable to reduce human effort. It is possible to find optimistic popular science articles dedicated to this topic on the internet (e.g., [7]). The use of such a simplified process would be welcome in many applications where it is difficult to stabilize GCPs, such as monitoring changes in the morphology of a volcano [8], landslides ([9,10]), dam and riverbed erosion [11,12,13], slow landslides [14], the risks associated with surface mining [15], slope stability in the vicinity of railways [16], the speed of glacier movement [17,18], or documenting rock outcrops [19]. This issue has been investigated by several authors who tested the accuracy of fixed-wing UAVs [20] with a higher travelling speed and working altitude and large custom-made UAVs, among others [21]. The novel testing of custom-made UAVs was published in [22,23] utilizing differential GPS (Global Positioning System) positions with the accuracy in decimeters. In [24], a digital surface model (DSM) was derived from the data acquired by a fixed-wing UAV SenseFly eBee-RTK, equipped with a compact camera Sony Cyber-Shot DSC-WX220 (18.2 Mpixel; focal length 4.5 mm). The latter study revealed that processing without the use of GCPs is problematic, particularly for the vertical component of the resulting model; thus, it is advisable to use at least one GCP set in the center of the area. In [25], the authors described a method for direct georeferencing without GCPs via the joint processing of data acquired by a quadrocopter flying at lower altitudes with data from a GNSS RTK-equipped fixed-wing UAV (senseFly eBee). The authors reported that their method was functional, although the results were again poorer for the vertical component and improved by adding at least a small number of GCPs. They also used pre-calibration for determining the interior orientation parameters directly on site, which, when used, improved the accuracy of (in particular) the vertical component (an approximately 1.5 (Ground sampling distance) GSD, compared to the original of approximately 3 GSD). 

On the other hand, the authors in [26] stated that “the RTK/PPK method of the georeferencing can provide data with comparable or even higher accuracy compared to the GCP approaches, independently on the terrestrial measurements”. The image acquisition was, similar to the previous study, performed using the senseFly eBee RTK, and all results derived solely through RTK georeferencing were better than the results derived using GCPs. In [27], the authors evaluated the performance of three software solutions (Agisoft PhotoScan, Pix4D, and MicMac) for the SfM processing of senseFly eBee RTK-acquired imagery, including testing the effect of the number of GCPs and their locations. The use of GCPs led to somewhat better results in the horizontal and approximately 50% improvement in the vertical components compared to the purely RTK-georeferenced data. The influence of the interior orientation parameters was also investigated, with the authors concluding that the “on-the-job calibration is likely in our opinion to be the best operational compromise, with the awareness that unless at least one GCP is provided, there might be biases in object coordinates, especially in elevation.” 

In [28], the effect of the trajectory of the data acquisition flight on the quality of the resulting DSM was studied along with the effect of the on-board GNSS RTK receiver recording camera positions. The data were acquired using a Mavinci Sirius Pro fixed-wing UAV mounted with a Panasonic LumixGX1-Pancake14mm-PRO camera. The authors demonstrated that a cross-flight pattern is beneficial for the quality of the resulting models and that the use of an on-board RTK receiver allows for accuracy similar to that achieved using GCPs but with a caveat regarding the need for a reasonable verification of the results. 

A similar experiment was performed in [29], where the georeferencing of a topographical model using the on-board (GNSS) receiver and an inertial measurement unit without GCPs was assessed. This study compared photogrammetric models georeferenced using (a) a UAV equipped with a GNSS RTK receiver, (b) the same UAV mounted with a GNSS receiver only, and (c) independently measured GCPs only. The authors concluded that the horizontal accuracy of the RTK UAV data processed by direct georeferencing was equivalent to the horizontal accuracy of the non-RTK UAV data processed with GCPs, but the vertical error of the DSM from the RTK UAV data was 2 to 3 times greater than the DSM from the non-RTK data with GCPs.

Direct georeferencing using GNSS RTK was also studied, for example, in [30], where no problems or systematic shifts/errors were recorded, and in [31], where, conversely, the risk of developing a systematic shift in the horizontal direction was reported.

The results reported so far are, therefore, not entirely consistent and usually concern fixed-wing UAV data that move different from rotary-wing UAVs (in their speed, motion character, and stability). Further, residual systematic shifts due to the synchronization between GNSS RTK and camera records can occur, as noted in [28]. In this study, we tested the first suitable low-cost complex commercial solution for rotary-wing UAVs—the Phantom 4 RTK. Given the results reported for this UAV (e.g., in [25,27,32,33]), we particularly focused on the vertical component. 

This experiment aimed to determine if it is possible to use only coordinates acquired by the UAV-mounted RTK receiver for processing and georeferencing (and if so, what the accuracy is) and what algorithms or processing methods can be used to improve the results. Two sites of different land types were used for testing—a mostly monochromatic homogenous area partially covered by tall woody vegetations (Rural) and a colorful broken (Urban) terrain with many buildings. Both of these areas were subject to three independent recordings to enable an assessment of the differences between individual image acquisitions for individual areas.

## 2. Materials and Methods

### 2.1. Data Acquisition

For the experiment, two areas of different characters from the perspective of tie point identification were used, each of them approximately 300 m × 300 m (90,000 m^2^) in size. The expected flight altitude was 110 m. The extent, flight altitude, and trajectory were designed to allow image acquisition of the whole area during a single flight (limited by battery capacity) and to yield the expected resulting point cloud accuracy comparable to the GNSS RTK measurement accuracy (approximately 0.03 m in the horizontal and 0.04 m in the vertical direction). 

According to [34], a horizontal accuracy of 0.5–1.0 GSD and a vertical accuracy of 1.5–2 GSD are generally achievable, corresponding to a flight altitude of approximately 100 m and a pixel size of approximately 0.03 m. On the other hand, the accuracy evaluation of a DEM (digital elevation model) for problematic terrain in [35] revealed significantly inferior vertical accuracy. 

**Figure 1:**
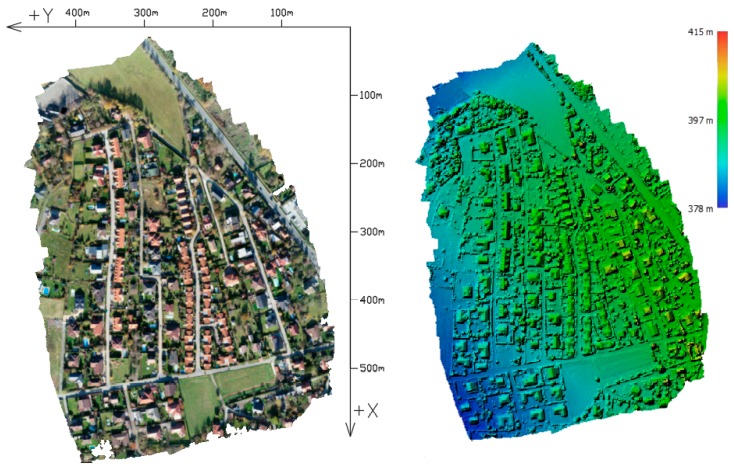
Urban area: orthophoto and DEM (Digital Elevation Model).

**Figure 2:**
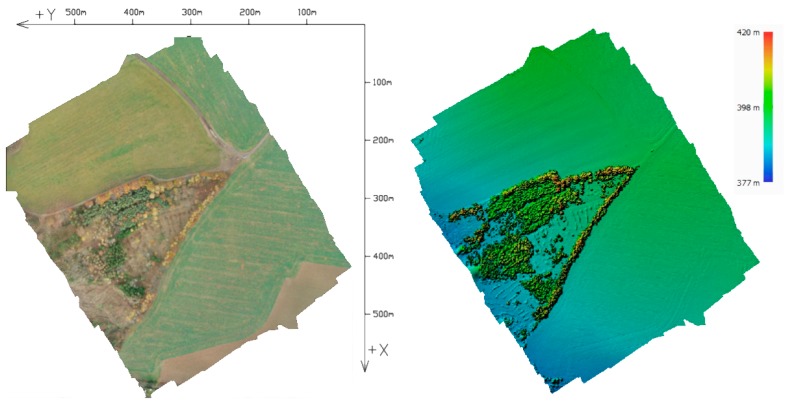
Rural area: orthophoto and DEM.

The first area of interest, the “Urban” area, is a built-up area with roads, houses, and greenery, featuring a surface with varied elevations and colors. This facilitates high-quality identification of tie points and, therefore, generally provides more reliable results than can be expected in the alternative area (see below; Figure 1). For the alternative area, an area with difficult-to-identify tie points was intentionally chosen (Figure 2). This area consists of homogenous surfaces—a field with low vegetation, grass, and a partial representation of tall and dense broadleaf trees (Rural).

**Figure 3:**
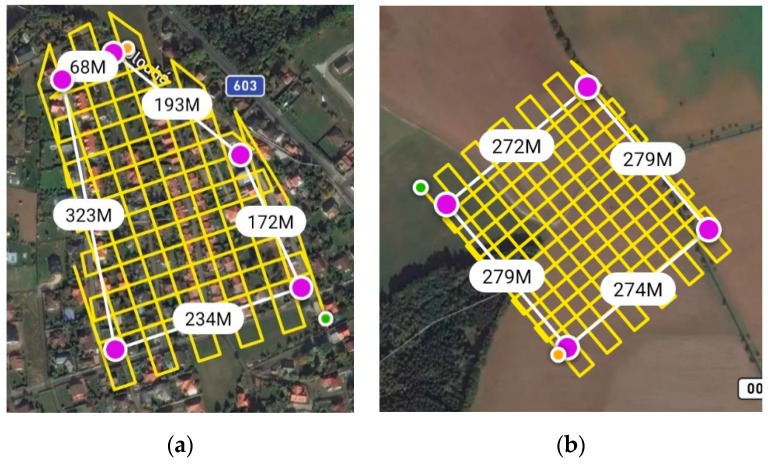
Flight trajectory for the Urban (**a**) and Rural (**b**) areas including distances.

For image acquisition, a UAV DJI Phantom 4 RTK mounted with a camera with a FC6310R lens (f = 8.8 mm), a resolution of 4864 × 3648 pixels, and a pixel size of 2.61 × 2.61 μm (price approximately 6000 Eur) was used. Figure 3 shows the trajectory of the image acquisition flights, including the dimensions; the image acquisition axis was always vertical (perpendicular to the flight axis).

Three independent flights using the same flight/image acquisition settings were performed (the same trajectory with a 75% forward overlap and a 75% sidelap at the ground level) at various times of the day (with approximately 2 h intervals; for the dates and times of the flights, see Table 1). Since the experiment sought to identify the problems of direct georeferencing using GNSS RTK, it was necessary to ensure that the impact of other circumstances of measurement was minimized. Agisoft Metashape has a special "Reduce overlap" function to determine the needed overlap of images. This was, in our case, 60% (using the high overlap option) in both directions. A 75% overlap in both directions was used to maximize the reliability of the results. The number of images was always 400 per flight. The GNSS RTK receiver was connected into the CZEPOS network of the permanent reference stations.

In addition to the image acquisition itself, a geodetic survey of ground control points (GCPs) and checkpoints was performed using a GNSS RTK Trimble Geo XR receiver with a Zephyr 2 antenna connected into the CZEPOS network of the permanent reference stations (czepos.cuzk.cz). The ground control points were marked using portable black and white targets (stabilized using a single ten-centimeter-long nail) with a diameter of 80 cm or a white painted cross 80 cm in size on the tarmac.

**Figure 4:**
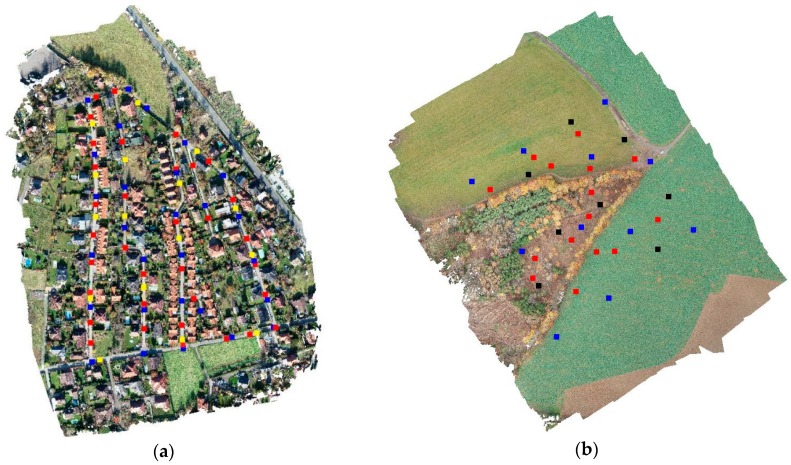
Placement of the ground control points (ground control points (GCPs), blue) and checkpoints (horizontal—black/yellow; vertical—red) for both sites – Urban (**a**), Rural (**b**).

Two types of checkpoints (CPs) were used—one for verification of the horizontal accuracy and one for verification of the altitudinal accuracy. In the Urban area, the CPs present in the landscape that were clearly identifiable on the resulting georeferenced orthophoto were selected (considering the expected image resolution of 0.03 m, the checkpoint size had to be at least 15 cm long to display the image on at least five pixels). In the Rural area, the targets had to be used as checkpoints as there were practically no naturally occurring and easily identifiable points in the area. The CPs for assessment of the vertical accuracy were, on the other hand, placed in a way that ensured they would be located on a flatter surface, so the assessment could be unambiguous and less dependent on the fact that the elevations of individual CPs were compared to the surfaces constructed from the individual points of the point cloud. The number of individual points in both study areas is detailed in Table 2, for the location of the points see the Figure 4.

### 2.2. Data Processing

The GNSS receiver measurements were exported from the WGS 84 coordinate system (latitude, longitude, ellipsoidal height). The spatial position in the same coordinate system was also extracted from the images (containing GNSS RTK data) using the Exiftool utility. All data were converted into the Czech national coordinate positioning system (S-JTSK, System of Unified Trigonometric Cadastral Network) and the Bpv; vertical datum (Balt after adjustment) using the EasyTransform software (http://adjustsolutions.cz/easytransform/) to ensure the same algorithm was used on all data and thereby eliminate potential systematic errors that could occur as a result of different transformation algorithms.

The image processing was performed via the Agisoft Metashape 1.6.1 software with the Structure from Motion calculation method (SfM) using the non-default settings listed in Table 3:

As the UAV was not equipped with a professional metric camera, the interior orientation parameters were determined via calculations in the usual way. Although pre-calibration is generally recommended, the stability of the parameters of the UAV-mounted camera were not fully ascertained. Other studies also reported that the use of laboratory calibration can be even less [36] or equally [37] accurate in comparison to the method used in this study. 

For each study area, the sparse point cloud was calculated in Agisoft Metashape, followed by the dense point cloud, and a DEM; based on this, an orthophoto with georeferencing was created. 

As three flights were performed on each site, three sets of measurements were produced for each area of interest. For each of set, three variants of the calculations were performed: the “RTK” variant, “GCP” variant, and “Combined” variant. In the RTK variant, the calculations were performed using only the UAV-recorded camera coordinates with a 0.03 m accuracy setting. In the GCP variant, the calculation used the GCPs with the preset 0.03 m accuracy; the camera coordinates were kept in the calculation, but their accuracy was set to 104 m. This ensured that their impact on the calculation was negligible but that the differences between the measured RTK positions and the calculated positions could still be derived. The Combined variant used both the GCPs and camera coordinates with an accuracy of 0.03 m (the 0.03 m accuracy was used because this number represents the root mean square of the standard deviations of two horizontal and one vertical component of the GNSS RTK). 

**Figure 5:**
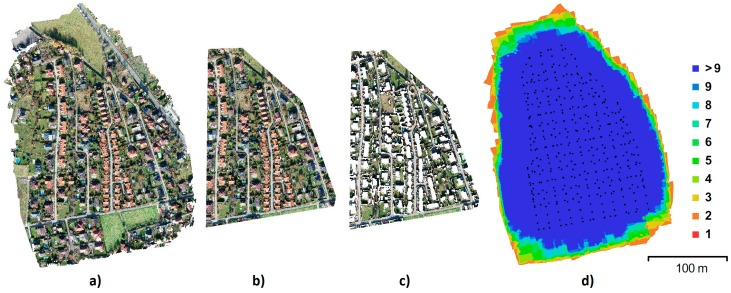
Urban data (**a**) original; (**b**) after cropping; (**c**) after ground filtering; (**d**) image coverage, black dots represent camera positions, and color hypsometry shows the number of overlapping frames.

**Figure 6:**
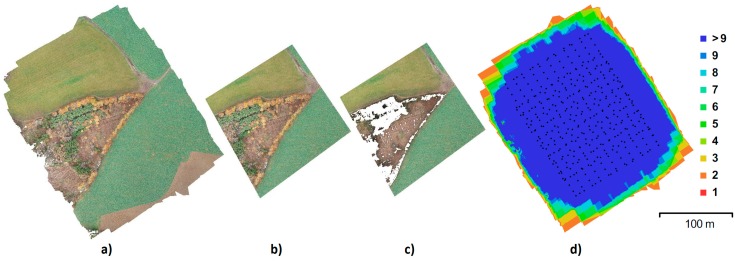
Rural data (**a**) original; (**b**) after cropping; (**c**) after ground filtering; (**d**) image coverage, black dots represent camera positions, and color hypsometry shows the number of overlapping frames.

The results of the calculations, therefore, always represent areas of 91,600 m^2^ with approximately 58 mil. points for the Urban area and 82,800 m^2^ with approximately 39 mil points for the Rural area. The originally calculated point cloud was always cropped to frame the area with GCPs to prevent undesirable deformation in the model edges. These data were subsequently ground filtered (using the CSF plugin in CloudCompare software with the resolution set to 0.5 m and the classification threshold set to 0.5), and only ground points were used for further comparisons. Figure 5 and Figure 6 show the individual variants and the coverage of the area by the imagery.

### 2.3. Accuracy Assessment

The data accuracy was tested in various ways. First, the data from the checkpoints and GCPs using ground-survey GNSS receivers were evaluated, and the accuracy from three repeats of the measurements was assessed. Secondly, the accuracy characteristics available directly in the Agisoft software, providing information on the internal accuracy of the model, were analyzed. Subsequently, the results were compared to independent measurements, and, lastly, the resulting point clouds were mutually compared to identify local deformations. To preserve the clarity of the text (i.e., to maintain the link between individual methods of calculation and their results), the details of individual analyses are provided together with the results of these methods.

## 3. Results

### 3.1. The Accuracy of the GNSS RTK Geodetic Survey

The accuracy of the measurements of GCPs and CPs was determined with a geodetic GNSS RTK receiver. Each point was measured three times at approximately 2 h intervals (similar to the flights). Table 4 details the standard deviations from the repeated measurements and confirms the consistent accuracy in both areas. It also shows that the achieved accuracies of the individual components were better than those expected before the experiment.

### 3.2. Internal Model Accuracy

While processing, Agisoft Metashape displays data for evaluation that can be used to analyze the internal calculation accuracy, such as the degree to which the coordinates acquired based on GCPs and those based on the UAV-mounted GNSS RTK mutually correspond. Without an additional (independent) verification, this is, in principle, the only information typically available to the user (however, this information does not provide the actual accuracy values).

Table 5 summarizes the accuracy characteristics calculated in Agisoft Metashape (root mean square deviations, RMSDs) and those based on its results (mean differences).

For the detection of systematic errors, the arithmetic mean differences between the measured and calculated positions were also determined (if no systematic shift and only random errors are present, the mean difference should be equal to zero). The RMSD of the GCP positions in the GCP calculation variant represents the internal measurement accuracy. The deviations are significantly higher in the vertical component and are worse than the expected accuracy of the UAV-mounted GNSS RTK receiver. It is also necessary to mention the deviation in the X coordinate in Flight 2 in the Urban area, the deviation of which is also approximately two times higher than that of the other flights. In the “GCP” calculation variant, the GCP mean differences are obviously always equal to zero (as the model is always placed in the center of the GCPs). The values of these mean differences for cameras represent, in this calculation, variants of the systematic shifts of all camera positions. The fact that these values are close to those of the RMSD implies that a major part of the RMSD is caused by the systematic shift of the camera positions.

The RTK calculation variant (based solely on the RTK camera positions) showed similar results. That the GCP deviations could not have been calculated as a simple test shows us that the presence (i.e., tagging) of GCPs in the images in the Agisoft software affects—albeit to a small degree—the results (even if tagged only as checkpoints with small accuracy). Therefore, the GCPs were not present at all in the calculations when processing this variant. The derived RMSD values are very small and do not indicate any problems. 

In the Combined calculation variant, the same accuracy (and therefore weight) was assigned to both the GCP and camera position coordinates. All resulting values show that although there were differences within the previous calculation variants, the measurements of both RTK camera positions and GCPs are correct, and the internal consistency of this model is better than the expected accuracy of the GNSS RTK measurement. The only exception is represented by the X coordinates of the second flight, where a major systematic shift between the on-board GNSS RTK and the terrestrial GNSS RTK receiver was observed. Its sizes are similar to those of the GCP variant. The presence of a shift in both calculations indicates that this systematic error was most likely caused by the GNSS RTK of the UAV receiver.

Table 6 shows the results of the identical calculations for the other area (Rural), the character of which entails a more difficult identification of tie points. For evaluation, it was also necessary to set a lower tie point accuracy (from the default value of 1 to 3.0 for the first flight, 3.5 for the second, and 4.5 for the third), as the coordinate differences after bundle adjustment were extremely high when using default values. Namely, the Total Error of the camera positions in the first flight was 2.129 m (values of 0.127 m; 0.113 m; 2.122 m for individual coordinates of X, Y, Z respectively), in the second flight, it was 0.535 m (0.109 m; 0.124 m; 0.509 m), and in the third flight, the Total Error was 0.632 m (0.096 m; 0.104; 0.613 m).

Comparing the GCP” calculation variant with the Urban area, it is obvious that the camera position differences in the Rural area are greater than those in the Urban area (as high as tens of centimeters in the vertical component). The arithmetic mean differences indicate that they are to a major degree caused by systematic shifts in all camera positions.

The calculations of the RTK variant revealed only small deviations not exceeding the expected measurement accuracy. As the GCPs were removed from the analysis, just like for the Urban area, their accuracy could not be determined. The mean shifts in camera coordinates are always equal to zero, as the model was fit to them. The “Combined” calculation variant with equal weights for GCPs and camera positions again shows very good consistency, which suggests that the measurements themselves are correct.

Overall, the accuracy is clearly better in the Urban area where the conditions for the identification of tie points are much better than those in the Rural area. This accuracy is on par with the GNSS accuracy, with the only exception represented by Flight 2 in the Urban area, which was burdened with a systematic X coordinate shift. Although the corrections of the recorded camera positions in the GCP calculation variant can be relatively large (compared to the assumed accuracy), these corrections may not necessarily be caused by measurement errors (as is obvious from the results of the Combined calculation). In this case, the probable reason is instead computational instability (see the Discussion). 

### 3.3. Verification Using Independent Checkpoints—Horizontal

To determine the absolute accuracy of the model, a comparison with an independent measurement was performed. 

The positions of the horizontal checkpoints were derived from the georeferenced orthophoto in the Bentley Microstation software (ver. 8.11) and were compared to the coordinates determined by a ground survey to acquire the accuracy characteristics shown in Table 7, namely the mean error, standard deviation, and RMSD for the X and Y coordinates. Table 7 also presents the mean values for the individual calculation methods, individual areas, and an overall mean including both study areas. The numbers in brackets, where shown, indicate the means after leaving out Flight 2 in the Urban area with the obvious systematic shift in the axis.

The verification of the horizontal accuracy shows equal accuracy for all calculation methods and confirms the systematic error of 0.08 in the RTK measurement of the UAV-mounted GNSS receiver in Flight 2 in the Urban area (see Table 7). This error did not occur in any of the other flights. However, regardless of whether it was caused by an error in the UAV itself, by the reference station network, or by any other source, it confirms the need to perform independent verification as is usual in geodesy (one measurement is no measurement), without which such an error would pass undetected.

### 3.4. Verification Using Independent Checkpoints—Vertical

The vertical checkpoints were compared with the point clouds of the respective calculation variants using only the vertical component of the distance. This comparison was performed in the CloudCompare software (www.cloudcompare.org). 

The point clouds were directly compared with the checkpoints by determining the minimum distance of each point from the point cloud to an irregular triangular network formed between the nearest twelve points of the SfM cloud (function Cloud to Cloud, tab Local modelling, and Local model option 2D1/2 Triangulation). The average vertical differences (systematic shift) and the standard deviations of the differences were also calculated and are shown in Table 8. 

In the vertical component, the RTK calculation variant shows substantial errors, namely a systematic vertical shift of the entire model by up to 0.14 m in the Rural area. In the Urban area (when leaving out the problematic second flight), the results are better, and the standard deviations meet expectations. The results of the “GCP” and “Combined” variants are similar and correspond to expectations (considering the accuracy of the checkpoint measurement and pixel size).

### 3.5. Comparison of Dense Clouds

When evaluating the results, it is also necessary to mutually compare the point clouds resulting from individual methods of calculation as the previous verifications were performed using isolated points only. Due to the point density, the comparison of dense clouds can be practically considered as continuous. Only the vertical component of ground filtered point clouds was compared here.

**Figure 7:**
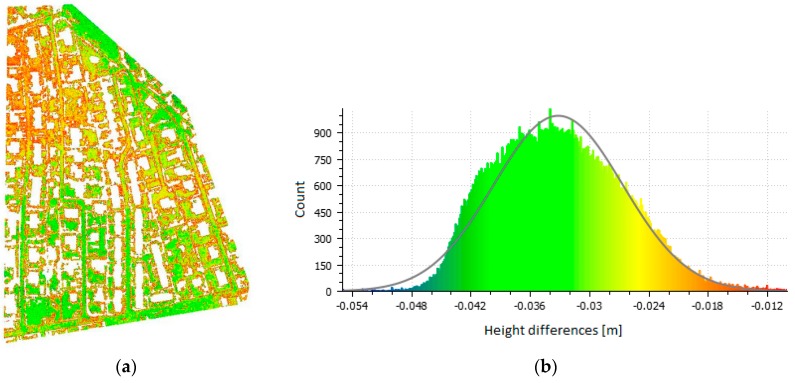
Point cloud comparison (**a**) (Combined vs. RTK, Urban area, Flight 1, and vertical deviations) and the histogram of errors (**b**).

**Figure 8:**
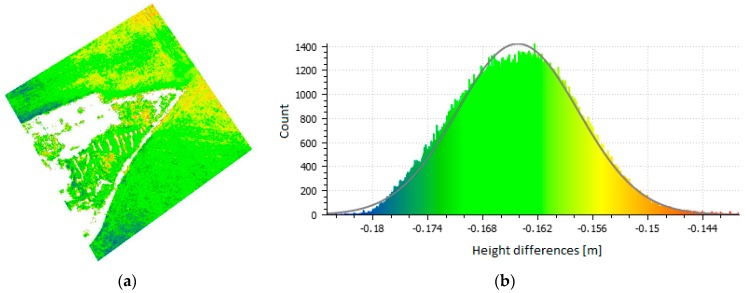
Point cloud comparison (**a**) (Combined vs. RTK, Rural area, Flight 1, and vertical deviations) and the histogram of errors (**b**).

An interesting and important fact is that the standard deviation is practically very similar in all variants and comparisons; the mean standard deviation is 0.22 m. The data (i.e., the entire photogrammetric models) are, therefore, mutually shifted solely in the vertical direction. Figure 7 and Figure 8 show the point clouds with a color scale indicating the sizes of the deviations and histograms showing the same color scale for Flight 1 in the Urban and Rural areas. The figures provide a comparison of the Combined variant (as the most accurate) with the RTK only variant (the one we were most interested in). The histograms show both the error distribution and systematic shift, confirming the conclusions from the checkpoints.

To compare the accuracy of the GCP and RTK, these variants were compared with the Combined variant (which was considered to be the most accurate) using a direct point cloud comparison (Table 9). 

Clearly, the RTK variant is always systematically shifted against both the Combined and GCP variants. In the Urban area, the systematic shift is approximately at the level of the ground sample distance (GSD; with the exception of the systematically shifted Flight 2); however, in the Rural area, it is significantly higher. When comparing identical calculation methods between individual flights (Table 10), the results are similar. In the RTK variant, the results for the Rural area are inferior to those for the Urban area. Otherwise, the standard deviations are practically equal to the GSD (ground sample distance), and the systematic errors are negligible when compared to the GSD.

## 4. Discussion

SfM-derived point clouds were constructed through multiple steps, including several decision and computation processes. It is, therefore, not possible to perform a simple estimation of the distribution of the measurement errors throughout computations or to derive the resulting accuracy only from the known measurement accuracy. Here, we used various data and processed them in several ways to show the differences that can result from different methods of calculation. 

To simplify the process of cloud point generation and, in effect, improve its speed and reduce the price, it would be ideal if the camera coordinates acquired from the on-board GNSS RTK receiver could yield a result of sufficient quality. Of course, the term “sufficient quality” is relative and depends on the application. Nevertheless, when using a UAV for modelling a relatively small area, it is reasonable to assume an accuracy of approximately 1–2 GSD. In our experiment, this was not the case. In the Urban area, the second flight was systematically shifted for unknown reasons, although that area was suitable for automatic SfM processing. This alone emphasizes the need for independent verification that can easily detect such major errors. A shift can also occur in the vertical component of the model as shown in the Rural area. In that case, however, the reason was not a serious systematic error like with the previous issue; instead, this error had its roots in the imperfect automatic optimization of a principally correct calculation. Table 5 and Table 6 show that without the use of independent verification (i.e., if only the RTK calculation based solely on the camera positions is used), there are no indications that anything is wrong. As the model is not deformed in any way in the RTKvariant (see Figure 7 and Figure 8) but only vertically shifted (these results are different from those reported in [38]), we also tested the influence of a small number of GCPs on the results. In all cases, even a single GCP removed any systematic shift. 

Flight 1 in the Rural area with a systematic vertical shift of 0.14 m (see Table 8) can serve as an example. After adding a single GCP, even in an area unsuitable from a configuration perspective, (i.e., in the lower right corner of the study area), the model was positioned more-or-less correctly (the systematic vertical shift dropped to 0.020 m, with a standard deviation of 0.026 m). We tested various positions for the individual GCPs, achieving practically identical results for all. Of course, we must bear in mind the principles of geodesy and always recommend multiple ground control points, at least in the four corners of the captured area.

The lack of accuracy in the vertical component in the rural area was likely due to the camera’s self-calibration process, which may have led to the miscalculation of internal camera parameters, as observed in [25] and [27]. When utilising non-metric cameras, bundle adjustment is usually calculated together with a calculation of the internal orientation elements, which, in most cases, yields quality results when using GCPs. Here, for the Rural area (difficult for SfM processing from the perspective of identification of tie points), this method is not suitable, as the bundle adjustment did not yield correct interior orientation parameters, which led to a vertical shift of the model. 

Table 11 shows that the calibrated focal lengths in the Combined calculation are very consistent while those of the RTK variant and provide results with much higher differences. We must, however, bear in mind that these values cannot be used as an unambiguous indicator as the interior orientation parameters are mutually dependent and cannot be evaluated separately. Further, the calibrated focal lengths are not consistent in the GCP variant, which otherwise provides correct results. The most reliable results (for the Combined calculation) are in bold, while italics denote the flight using a systematic shift. The underlined italics highlight the most outlying results (Rural RTK calculations).

For these reasons, we also performed an RTK calculation variant using calibration parameters derived from the Combined variant from the same flight, and the results were practically of the same quality as those from the Combined calculation (the mean vertical shifts were similar to those yielded using the Combined variant). This was true for all flights in both areas, with the exception of Flight 2 in the Urban area (the one with the systematic X-axis shift). This proves that the inferior results in the Rural area were likely caused by the incorrect determination of the interior orientation parameters during the calculation. In other words, inferior quality tie points make problematize the correct optimization of the interior parameters, which leads to inferior results. In the UAVs equipped with metric cameras where the stability of the interior orientation parameters can be expected, quality pre-calibration should, therefore, be able to overcome this problem and allow the production of quality models even without the use of GCPs. However, we still recommend to use some independent verification (e.g., a small number of “checkpoints”) as there is always a risk of an error, such as that for Flight 2 of the Urban area, where the GNSS RTK receiver systematically determined the X coordinate erroneously in all images, no matter the cause. 

Comparing our results achieved using a quadrocopter and those reported by [24], who used a fixed-wing UAV, we conclude that the expected improvement of the data accuracy based solely on the onboard GNSS RTK data (expected due to the lower travelling speed and better stability) was not confirmed.

## 5. Conclusions

Affordable GNSS-RTK equipped multicopters have only recently become available. Their lower traveling speed and higher stability (compared to fixed-wing UAVs) suggests that they could be more suitable for the direct processing of acquired imagery without the need for GCPs; this was the principal hypothesis of this study. Our results, however, prove that the use of a GNSS RTK receiver in a multicopter UAV without external verification is potentially very dangerous—a danger manifested predominantly as a systematic shift of the entire point cloud in the vertical components. The horizontal components (unless a systematic error of the UAV positioning system occurred) were practically unaffected. 

To the best of our knowledge, we are the first to use two separate testing sites with different characteristics (Urban and Rural) for the evaluation of the suitability of GNSS RTK-equipped UAVs (fixed-wing or multicopter) for the production of models without GCPs. This allowed us to perform an in-depth comparison of the results and to analyse the reasons for their differences. The finding that the vertical error increases with the declining suitability of the terrain for SfM processing (even when using a GNSS-RTK-mounted multicopter) is not surprising but remains important for the practical application of this type of UAV.

Further testing, however, showed that this problem can be overcome in two ways. The first involves using a small number of ground control points (in a small area, such as those used in our study, as even a single GCP anywhere in the area was sufficient to rectify the problem). The other method is potentially more important for future practice. We showed that when using an optimum set of calibration parameters for a non-metric camera (reversely derived from the best calculation variant), one can achieve extremely accurate results, even when using GNSS RTK data only. This suggests that the use of metric cameras that can be perfectly pre-calibrated could also provide excellent results, even without the use of GCPs for the production of the model (note, however, that this model still needs to be verified externally afterwards to prevent shifts such as that in Flight 2 in the Urban area).

We also conclude that if GCPs are used, the horizontal and vertical accuracy will be practically identical to the GSD (here it is 0.028 m; it is also necessary to consider the accuracy of the verification method). This provides the safest method for data acquisition and processing. Moreover, the best model resulted from a combination of both GCPs and GNSS RTK UAV coordinates for the calculations.

## Figures and Tables

**Table 1:** Dates and times of the image acquisition flights.

Flight	Urban-7.11.2019	Rural-22.11.2019
1	11:08 to 11:27 h	11:15 to 11:34 h
2	13:11 to 13:29 h	13:28 to 13:46 h
3	15:02 to 15:20 h	15:22 to 15:41 h

**Table 2:** The numbers of GCPs and control points (CPs) in the individual study areas.

	GCPs	CPs–Horizontal	CPs–Vertical
Urban	28	38	36
Rural	11	8	15

**Table 3:** Agisoft Metashape software settings used for calculations.

Setting	Value
Align Photos	
ccuracy	high
Key point limit	40,000
Tie point limit	4000
Optimize Camera Alignment	fit all constants (f, cx, cy, k1–k4, p1–p4)
Build Dense Cloud	
Quality	High
Depth filtering	Moderate
DEM	
Projection	Geographic
Parameters	
Source data	Dense Cloud
Interpolation	Enabled
Advanced	
Resolution	2.8 cm/pix (implicit)
(Settings not detailed above were kept at default).	

**Table 4:** Standard deviations of the coordinates of the GCPs.

Area	S_x_ [m]	S_y_ [m]	S_z_ [m]
Urban	0.008	0.008	0.013
Rural	0.009	0.008	0.014

**Table 5:** Results for the Urban area—RMSDs and the mean differences between the calculated and measured camera/GCP positions.

Variant	Flight	Diff. on	RMSD			Mean Differences		
			X[m]	Y[m]	Z[m]	X[m]	Y[m]	Z[m]
GCP	1	Cameras	0.049	0.044	**0.246**	−0.000	−0.006	**0.245**
		GCPs	0.009	0.011	0.021	0	0	0
	2	Cameras	**0.086**	0.050	**0.222**	**−0.066**	0.016	**0.220**
		GCPs	0.009	0.010	0.025	0	0	0
	3	Cameras	0.055	0.051	**0.086**	−0.004	−0.016	**0.084**
		GCPs	0.009	0.010	0.020	0	0	0
RTK	1	Cameras	0.008	0.007	0.012	0	0	0
		GCPs	-	-	-	-	-	-
	2	Cameras	0.007	0.007	0.008	0	0	0
		GCPs	-	-	-	-	-	-
	3	Cameras	0.007	0.007	0.008	0	0	0
		GCPs	-	-	-	-	-	-
Combined	1	Cameras	0.017	0.018	0.013	0.001	0.000	0.000
		GCPs	0.012	0.012	0.021	−0.008	−0.001	−0.003
	2	Cameras	0.018	0.016	0.012	−0.005	0.001	0.000
		GCPs	0.064	0.020	0.016	0.064	−0.017	0.000
	3	Cameras	0.018	0.019	0.010	0.000	−0.001	0.000
		GCPs	0.009	0.013	0.017	−0.003	0.008	−0.002

**Table 6:** Results for the Rural area—RMSDs and mean differences.

Variant	Flight	Diff. on	RMSD			Mean Difference		
			X [m]	Y [m]	Z [m]	X [m]	Y [m]	Z [m]
GCP	1	Cameras	0.035	0.026	**0.335**	0.010	0.006	**0.334**
		GCPs	0.003	0.004	0.015	0	0	0
	2	Cameras	0.028	0.031	**0.303**	0.013	0.007	**0.302**
		GCPs	0.005	0.004	0.011	0	0	0
	3	Cameras	0.026	0.029	0.016	0.006	−0.011	−0.006
		GCPs	0.005	0.006	0.007	0	0	0
RTK	1	Cameras	0.006	0.006	0.012	0	0	0
		GCPs	-	-	-	-	-	-
	2	Cameras	0.004	0.004	0.016	0	0	0
		GCPs	-	-	-	-	-	-
	3	Cameras	0.001	0.002	0.004	0	0	0
		GCPs	-	-	-	-	-	-
Combined	1	Cameras	0.012	0.012	0.013	0	0	0
		GCPs	0.008	0.007	0.010	0.003	0.003	−0.001
	2	Cameras	0.011	0.011	0.017	0	0	0
		GCPs	0.015	0.013	0.011	−0.009	−0.002	0.000
	3	Cameras	0.019	0.020	0.014	0	0	0
		GCPs	0.008	0.011	0.014	−0.004	0.009	0.005

**Table 7:** Horizontal checkpoint accuracy indicators.

Calculation Variant	Area	Flight	Mean Deviation		Standard Deviation		RMSD	
			X [m]	Y [m]	X [m]	Y [m]	X [m]	Y [m]
RTK	Urban	1	0.020	0.005	0.019	0.019	0.021	0.020
		2	0.082	−0.023	0.016	0.015	0.083	0.027
		3	0.005	0.006	0.015	0.016	0.016	0.017
	Rural	1	0.004	0.006	0.020	0.023	0.019	0.023
		2	0.001	0.002	0.022	0.025	0.020	0.024
		3	0.009	0.006	0.019	0.024	0.020	0.024
	**Mean**		**0.020 (0.008)**	**0.000 (0.005)**	**0.019 (0.019)**	**0.021 (0.022)**	**0.038 (0.019)**	**0.023 (0.024)**
GCPs	Urban	1	0.005	0.004	0.018	0.022	0.018	0.022
		2	0.012	−0.001	0.016	0.019	0.020	0.018
		3	0.008	0.004	0.020	0.023	0.021	0.023
	Rural	1	0.006	0.009	0.017	0.020	0.017	0.021
		2	0.007	0.012	0.022	0.026	0.022	0.027
		3	0.011	0.010	0.014	0.023	0.018	0.024
	**Mean**		**0.008**	**0.006**	**0.018**	**0.022**	**0.019**	**0.023**
Combined	Urban	1	−0.006	0.009	0.018	0.022	0.018	0.023
		2	0.072	−0.017	0.019	0.015	0.074	0.023
		3	0.006	0.008	0.021	0.020	0.021	0.021
	Rural	1	0.008	0.006	0.017	0.021	0.018	0.021
		2	0.002	0.007	0.025	0.024	0.024	0.024
		3	0.000	0.014	0.019	0.023	0.018	0.026
	**Mean**		**0.014 (0.002)**	**0.005 (0.009)**	**0.020 (0.020)**	**0.021 (0.022)**	**0.035 (0.020)**	**0.023 (0.023)**

**Table 8:** Results of the accuracy assessment of the vertical checkpoints.

Variant	Flight	Urban			Rural		
		Mean Deviation [m]	Standard Deviation [m]	RMSD [m]	Mean Deviation [m]	Standard Deviation [m]	RMSD [m]
RTK	1	0.029	0.021	0.036	**0.144**	0.032	**0.147**
	2	**−0.090**	**0.021**	**0.093**	0.041	0.028	0.049
	3	0.014	0.023	0.027	0.086	0.025	0.090
	**Mean**	**−0.016** **(0.022)**	**0.022** **(0.022)**	**0.060** **(0.032)**	**0.090**	**0.028**	**0.103**
GCP	1	−0.010	0.028	0.030	−0.034	0.028	0.043
	2	−0.024	0.030	0.039	−0.029	0.028	0.040
	3	−0.014	0.029	0.032	−0.012	0.023	0.025
	**Mean**	**−0.016**	**0.029**	**0.034**	**−0.025**	**0.026**	**0.037**
Combined	1	−0.009	0.022	0.024	−0.023	0.030	0.037
	2	−0.023	0.024	0.034	−0.017	0.027	0.031
	3	−0.013	0.024	0.027	−0.014	0.027	0.030
	**Mean**	**−0.015**	**0.023**	**0.029**	**−0.018**	**0.028**	**0.033**

**Table 9:** Comparison of the GCP and RTK variants with the Combined variant.

Flight	Variant Compared		Urban		Rural	
			Mean Deviation [m]	Standard Deviation [m]	Mean Deviation [m]	Standard Deviation [m]
1	Combined	GCP	0.003	0.020	0.004	0.015
		RTK	**−0.033**	0.012	**−0.163**	0.019
2	Combined	GCP	0.003	0.020	0.006	0.020
		RTK	**0.060**	0.019	**−0.056**	0.015
3	Combined	GCP	0.003	0.017	−0.002	0.020
		RTK	**−0.025**	0.012	**−0.093**	0.018

**Table 10:** Mutual comparison of the calculation variants.

Variant	Flight Compared		Urban		Rural	
			Mean Deviation [m]	Standard Deviation [m]	Mean Deviation [m]	Standard Deviation [m]
Combined	1	2	0.006	0.025	0.006	0.017
		3	0.003	0.027	−0.011	0.019
GCP	1	2	0.006	0.026	−0.005	0.017
		3	0.003	0.026	−0.016	0.021
RTK	1	2	**0.100**	0.037	**0.099**	0.021
		3	0.010	0.027	0.058	0.020

**Table 11:** Interior orientation parameters determined for the individual calculation variants and flights (ME–Mean error in the vertical direction).

Area	Flight	Variant	F/pix	Cx/pix	Cy/pix	ME/m
Urban	1	Combined	**3683.1015**	**7.9537**	**31.7125**	0.024
	2		*3687.0770*	*7.0952*	*26.9476*	0.034
	3		**3683.8230**	**7.9741**	**31.6299**	0.027
	1	RTK	3684.2895	7.6251	30.8493	0.036
	2		3684.5967	6.7025	27.5137	0.093
	3		3684.5744	7.6400	30.8406	0.027
	1	GCP	3691.6372	9.1263	29.8708	0.030
	2		3694.8683	8.5679	25.1348	0.039
	3		3687.0244	9.2974	31.7190	0.032
Rural	1	Combined	**3683.3930**	**7.1279**	**30.7098**	0.037
	2		**3682.1743**	**7.5854**	**29.3024**	0.031
	3		**3684.4672**	**7.9307**	**31.1895**	0.030
	1	RTK	*3691.0187*	*7.1598*	*28.3578*	*0.147*
	2		*3686.3828*	*7.5746*	*27.8308*	*0.049*
	3		*3688.1387*	*7.7183*	*29.8556*	*0.090*
	1	GCP	3693.9528	7.7744	26.9313	0.043
	2		3692.3433	8.0277	25.2495	0.040
	3		3684.2539	8.2464	31.1109	0.025
